# Colloidal Hollandite Holey Rods Produced by Presynthetic Nanohybridization

**DOI:** 10.1021/acs.nanolett.5c01451

**Published:** 2025-06-26

**Authors:** Ilenia Maria D’Angeli, Graziano Rilievo, Simone Molinari, Anna Barbaro, Alessandro Cecconello, Aura Cencini, Federica Tonolo, Mary Bortoluzzi, Marco Favero, Andrea Basagni, Sheryl Anne Singerling, Frank Eric Brenker, Fabio Vianello, Massimiliano Magro, Gabriella Salviulo

**Affiliations:** † Department of Geosciences, 9308 University of Padova, Via Gradenigo 6, 35131 Padova, Italy; ‡ Department of Comparative Biomedicine and Food Science, 9308 University of Padova, Viale dell’Università 16, 35020 Legnaro (PD), Italy; § Museum of Nature and Humankind, Mineralogy Section Alessandro Guastoni, 9308 University of Padova, Via Giotto 1, 35121 Padua, Italy; ∥ Schwiete Cosmochemistry Laboratory, Department of Geoscience, Goethe Univerity Frankfurt, Altenöferallee 1, 60438 Frankfurt, Germany; ⊥ Department of Molecular and Translational Medicine, University of Brescia, Viale Europa 11, 25123 Brescia, Italy; # Department of Chemical Sciences, 9308 University of Padova, Via Marzolo 1, 35131 Padova, Italy

**Keywords:** titanate nanotubes, SPIONs, nanoassembly, reagent confinement, ion exchange, nanomaterials

## Abstract

Electrostatically stabilized binary hybrids comprising TiO_2_ nanotubes and Fe_2_O_3_ nanoparticles were self-assembled and investigated as precursors for a KFTO material. Presynthetic nanohybridization is a way to organize the components, with the caveat that the mere nanomaterial combination cannot grant a high degree of control due to their general susceptibility to aggregation, resulting in masses with poor spatial order. Various hybridization conditions were explored, and the effects of the experimental parameters were investigated in detail, considering KCl concentration, Fe/Ti ratio, and hydrothermal treatment temperature. The optimized synthetic product was obtained at a remarkably low temperature (800 °C), and it was characterized by small size, partially hollow morphology (cavity diameter ca. 100 nm), and water colloidal stability, likely inherited from the parent nanotubes. These hollow rods can be envisioned as nanoreactors for confined space synthesis and as tools for environmental remediation.

## Graphical abstract

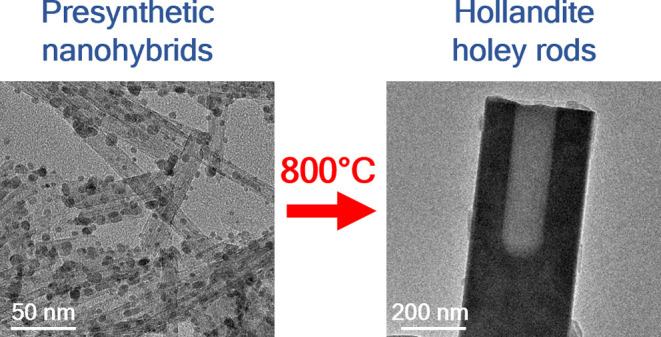

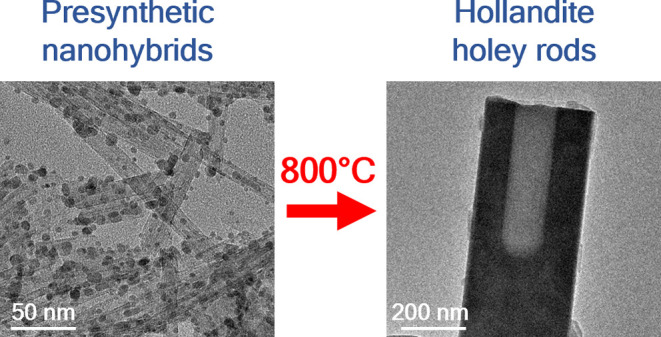

The opportunity to combine different nanomaterials into binary, or even more complex hybrid systems, has the potential of expanding the boundaries of nanotechnology, responding to the demand of tailoring, processing, and using functional nanostructures in critical application fields, such as nanobiomedicine,^1–5^ biosensors,^6,7^ quantum devices,^8^ and soil/water remediation.^9^ Regardless of the difficulties associated with multi-nanoparticle fabrication, the ability to custom-design these architectures would represent an advancement for their rapid in-field application.^10^ Among the most attractive properties of these materials there are those resulting from the collective interactions across the assembly nanocomponents and controlled by nanoparticle features, for example, secondary structure orientation phase, symmetry, or dimensions.^11–13^

Here, nanomaterial hybridization was used as a strategy for confining reagents at the nanosize level, and the effects of this approach on the formation of hollandite were compared to the synthetic route that employs bulk iron and titanium oxides as reagents.^14^ Recently, hollandite supergroup minerals attracted interest for their biological inertness, chemical stability, fast ion conduction, and ion exchange ability,^15,16^ which make them suitable adsorbents for radioactive elements or solid electrolytes.^17–21^ Moreover, hollandite-based semiconductors were widely employed for electrochemical energy storage.^22,23^

The stoichiometry of hollandite materials can be represented by the general formula A_*x*_B_8_O_16_, where A is an alkaline and/or alkaline-earth cation, while B is a metal such Al, Fe, Cr, or Ti, with varying oxidation states.^23^ Potassium titanates with a hollandite structure possess a high mobility of potassium ions in the channels of titanium–oxygen octahedra, but exhibit a low electrical conductivity (down to 10^–8^ S cm^–1^).^24,25^

In the burgeoning context of new functional material research,^26,27^ the current study focused on developing a titanium oxide derivative comprising potassium, iron, titanium, and oxygen (KFTO), namely, a Fe­(III)-doped potassium titanate commonly referred to as the hollandite supergroup.^28^ Indeed, different protocols were proposed for the synthesis of KFTO hollandite, which produced rods with various sizes, purities, and properties.^14,21,24,29–31^ In this view, the main challenge in developing novel materials is to control the nucleation and growth stages during the reaction.^32^

Herein, an electrostatically stabilized hybrid was self-assembled by a simple water incubation of oppositely charged TiO_2_ nanotubes (TiNTs) and surface active maghemite nanoparticles (SAMNs).

After its thermal treatment at 800 °C, a novel KFTO hollandite was obtained and thoroughly characterized by Fourier-transform infrared spectroscopy (FTIR), μ-Raman spectroscopy (MRS), atomic force microscopy (AFM), transmission electron microscopy (TEM), scanning transmission electron microscopy (STEM), dynamic light scattering (DLS), and X-ray powder diffraction (XRPD), showing a hollow cylindrical morphology, submicron size, and outstanding colloidal stability in water.

The synthesis presented in this work could expand the range of hollandite technological opportunities. In fact, besides the general properties that convey interest in these materials, synthetic hollow nano- and micromaterials represent elective platforms to develop nanoreactors. The latter have an enormous potential for a wide range of applications, from environment remediation to energy storage,^33^ mimicking the way nature fabricates functional compartments to perform complex biological processes. For example, compartmentalization for nanoreactor applications can be obtained by confining active chemical species into well-defined volumes through “postdecoration approaches” which consist in the internalization of a variety of metal particles (e.g., noble metal particles) in the preformed cavity while controlling their combination, number, and population density.^34^ Among such approaches, the “ship-in-a-bottle” process is the most representative, and it involves the growth of the incorporated precursors into larger nanocrystals through reduction or assembly reactions, so that the active material is captured inside the cavity.^35^ The combination of colloidal stability and nanosized volumes makes the holey submicron rods, proposed in this work, good candidates for the fabrication of nanoreactors through the “ship-in-a-bottle” strategy. In fact, while their stability in water enables them to carry out reactions in an aqueous milieu, the submicrometer size allows their separation by centrifugation from nanosized colloidal particle excess. The present results are expected to stimulate the use of nanomaterial hybrids as a precursor for novel materials with emerging properties.

On this rationale, electrostatically stabilized hybrids were developed by a self-assembly reaction, simply mixing colloidal suspensions of TiNTs and SAMNs, as described in the Materials and Methods section (see Supporting Information). Figure 1A–C illustrates the morphology of the TiNTs and SAMNs. TiNTs suspension’s zeta potential (ζ) was −37.9 ± 0.4 mV (conductivity = −0.053 mS cm^–1^ in water at 25 °C), while SAMNs displayed a positive ζ above +30 mV, as extensively reported elsewhere.^36^ The TiNTs anisotropic hydrodynamic size is reported in Figure S1A.

**Figure 1:**
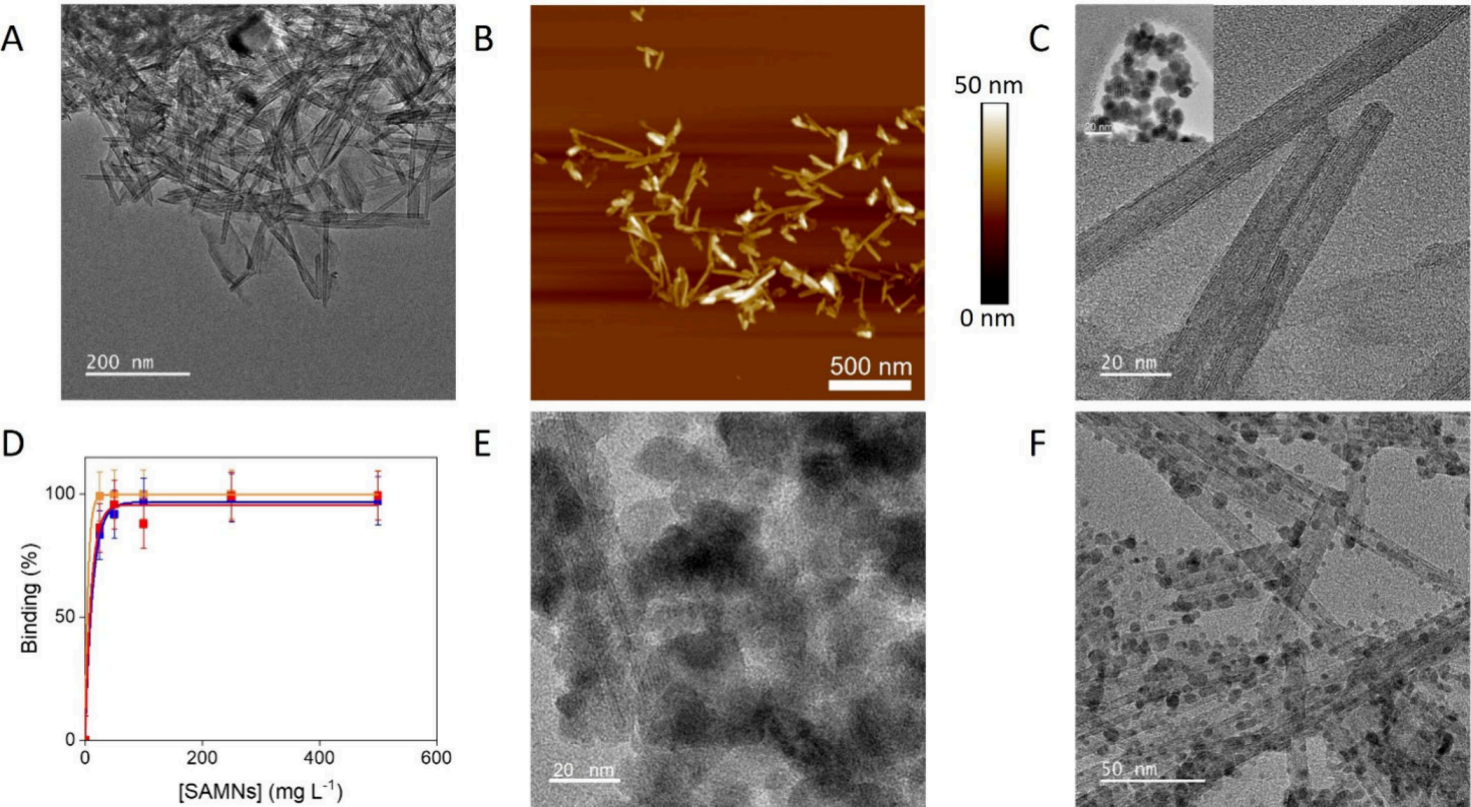
Self-assembly wet reaction between TiNTs and SAMNs. (A, B) TEM bright field (BF) and AFM images of parent TiNTs. (C) Higher magnification TEM-BF image showing TiNT multiwalled structure, inset: HR-TEM image of isolated SAMNs. (D) Percentage of magnetically captured TiNTs as a function of magnetic nanoparticle concentration and 10 mM (red line), 20 mM (blue line), or 50 mM (yellow line) KCl. Error bars represent standard deviations; measurements were performed in triplicate. (E) TEM-BF image of TiNTs-SAMNs hybrids obtained through the self-assembly reaction in the presence of an excess of maghemite and (F) TEM-BF image of TiNTs-SAMNs hybrids obtained from the incubation of 100 mg L^–1^ of TiNTs and 25 mg L^–1^ of SAMNs.

The electrostatic interaction between TiNTs and maghemite nanoparticles is expected to be pivotal to the development of the KFTO hollandite and, for this reason, potassium was assumed to have the double role of hybrid formation-promoting agent as well as an elemental component of the new synthetic material.

Optimal synthetic conditions were investigated by screening KCl concentrations in the range between 1 and 50 mM and varying the TiO_2_/Fe_2_O_3_ mass ratio (w/w) from 4 to 0.2. The binding efficiency as a function of SAMN and KCl concentrations after incubation and magnetic separation is shown in Figure 1D. Stability tests were conducted by repeatedly incubating the magnetically separated materials with fresh KCl solutions, where bound TiNTs were estimated as the difference between the initial and unbound nanotube concentrations by UV–vis absorption measurements, considering both the incubation and washing of supernatants (for detailed procedure descriptions and accompanying experimental results, see Materials and Methods).

At KCl concentrations below 10 mM, binding was completely reversible and the nanoconjugate separated into its parent components (Table S1), corroborating the assumed electrostatic nature of the interaction. Conversely, the loss of bound nanotubes was minimal when the ionic strength exceeded 10 mM KCl. The binding efficiency, expressed as the fraction of bound nanotubes, increased with nanoparticle concentration, following an exponential trend and reaching a plateau at approximately 25 mg L^–1^ of SAMNs (Figure 1D). The percentage of binding during the hybridization process is reported in Table S1. However, as the nanoparticle concentration was further increased, the occurrence of SAMN aggregation phenomena became progressively more relevant (Figure 1E). Thus, 25 mg L^–1^ of SAMNs is an ideal threshold at which colloidal coagulation is still hampered in favor of a more homogeneous distribution of single iron oxide nanoparticles onto the TiNT surface. Figure 1F shows a TEM-BF image of iron oxide nanoparticles nicely decorating the titanium oxide nanotube surface after incubation in 50 mM KCl.

After the binding process, samples were cured at 600 or 800 °C and subjected to XRPD analysis (Table S2, Figure S2). XRPD data of the different samples were analyzed to estimate the quantity of each phase. Samples were washed with ultrapure water to remove salts, including halite and sylvite, and then they underwent Rietveld refinement to obtain quantitative fractions of the different mineralogical species and to define crystal parameters.^37^

KFTO hollandite formation took place exclusively when (i) precursor incubation occurred at 50 mM KCl or higher and (ii) hybrids were cured at 800 °C. Scant traces of titanium-based minerals were generated in the crystalline form of freudenbergite (5%) and anatase (18%), along with a large amount of hematite (64%), sylvite (4%), unreacted maghemite (7%), and titanate nanotubes (2%) when precursors were incubated at 600 °C and 10 mM KCl (Figure S2A, Table S2). Figure S2B reports XRPD data showing that 50 mM KCl precursor incubation and 600 °C curing led to a large amount of sylvite (80%). When the thermal treatment was carried out at 10 mM KCl and 800 °C curing, the scenario was still characterized by a rather heterogeneous composition that included hematite (83%), anatase (11%), rutile (2%), freudenbergite (2%), and pseudobrookite (2%), Figure S2C. In contrast, when the process was performed at KCl concentrations equal to 50 mM, or higher, and 800 °C curing, KFTO hollandite formation occurred (Figure S2D and E). Along with the desired product, hematite and sylvite were present as two main byproducts. It can be assumed that the excess of SAMNs or KCl resulted in the increased percentage of these contaminants, at the expense of the KFTO hollandite purity.

KCl concentration increment to 250 mM offered no advantages in terms of KFTO hollandite phase enrichment, while it resulted in a drastic increase of the salt byproduct (Figure S2E). Thus, 50 mM KCl was identified as the optimal concentration. The minor components sylvite and halite were easily and completely removed by postsynthetic washing with ultrapure water. Hematite contribution was almost completely zeroed by using 25 mg L^–1^ SAMNs in the initial hybridization step (Figure S2F, Table S2). Figure 2A shows the trends of hematite and KFTO hollandite relative abundances as a function of the increasing TiNTs/SAMNs mass ratio. While hematite decreased, following an exponential decay, KFTO hollandite exhibited the opposite behavior and was well-fitted by an exponential growth with 100% yield at 25 mg L^–1^ of SAMNs. The XRPD pattern (Figure 2B) of the KFTO hollandite phase obtained using 50 mM KCl and the 4:1 TiNTs/SAMNs mass ratio was refined through Rietveld analysis, matching published peak positions and relative intensities.^14^

**Figure 2:**
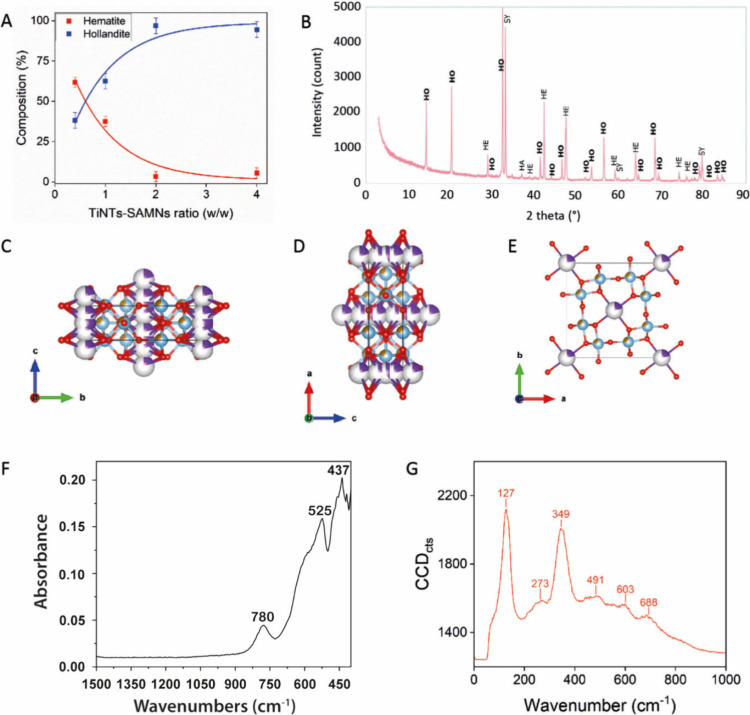
Synthesis optimization and chemical-physical characterization of the as-obtained high-purity product. (A) Fraction of KFTO hollandite *versus* hematite phase composition as a function of TiNTs/SAMNs ratio of the former electrostatically stabilized hybrid. Error bars represent standard deviations; measurements were performed in triplicate. (B) XRPD diffractogram of the sample characterized by 25 mg L^–1^ of SAMNs and 50 mM KCl after curing at 800 °C. HO refers to KFTO hollandite, HE to hematite, HA to halite, and SY to sylvite. (C–E) Three views of the unit cell of the synthetic KFTO hollandite, which were drawn by using Vesta software; (C) *a* axis vertical; (D) *b* axis vertical; (E) *c* axis vertical. Dots represent atoms: oxygen is red, titanium is light blue, iron is light brown, potassium is purple, and vacancies are white. (F, G) FTIR and Raman spectra, respectively, of the synthetic KFTO hollandite.

The results of the Rietveld refinements are presented in Table S3. The lattice parameters, the atomic positions, and occupancies were found to be consistent with values reported in JCPDS 98-019-2751,^14^ K_1.55_(Ti_6.5_Fe_0.1.5_)­O_16_, tetragonal crystal system *I*4/*m*; *a* = *b* = 1.01503(1) nm, *c* = 0.29717(1) nm (Table S3), while three views of a unit cell graphical representation of the synthetic KFTO hollandite are reported in Figure 2C, D, and E. FTIR and Raman spectra were collected to investigate the Fe–Ti–O bonds in the KFTO hollandite. FTIR peaks (Figure 2F) in the region 400–800 cm^–1^ were ascribed to (Fe/Ti)­O_6_^8–^ octahedral modes and Ti–O–Ti vibrations.^22,38^ In particular, the signal at 780 cm^–1^ was attributed to the stretching vibration of (Fe–Ti)­O_6_^8–^ octahedra,^38^ while peaks at 437 and 525 cm^–1^ were related to (Ti–O–Ti) bonds.^22^ Raman spectral analyses of KTFO hollandite (Figure 2G) displayed two sharp peaks centered at 127 and 349 cm^–1^ and four broad features at ∼273, ∼491, ∼603, and ∼688 cm^–1^ Raman shift. The peak at 127 cm^–1^ was attributed to the symmetric stretching of (Fe–Ti)­O_6_ octahedra,^39^ while the other above-mentioned features are the result of (Fe–Ti)­O_6_ octahedra bending modes.^40^

As a control, the synthetic product obtained by skipping the preliminary self-assembly reaction in 50 mM KCl and the 4:1 TiNTs/SAMNs mass ratio was analyzed by XRPD after curing at 800 °C, resulting in KFTO hollandite. In the absence of the initial electrostatic driven hybridization, the cured material, when simply vortexed in an aqueous solution, showed the emergence of a macroscopic particulate. This coarse component was resistant to a prolonged ultrasound treatment and, therefore, not analyzable by AFM or DLS. However, the zeta potential of the material present in the as-obtained supernatant was measured and it resulted to be −20 ± 6 mV (conductivity was 0.065 mS cm^–1^), characterizing this minor water dispersed fraction as a moderately stable suspension (−30 < ζ < −20).^41^ The correlation curve obtained by DLS analysis of the material, without the presynthetic hybridization, showed a very high polydispersity and an increase in the mean hydrodynamic size, as indicated by the high noise over 10^4^ μs (Figure S3, blue line). In Figure S4A, the overall aggregated nature of the material can be clearly appreciated by TEM micrographs, showing extended bundled masses in vast regions of the sample. These apparently anisotropic items are hardly distinguishable due to their entanglement and overall morphological disorder (Figure S4B and C). The situation is further exacerbated by the presence of a matrix, apparently merging the undefined objects together (Figure S4D). Irregular columnar shapes were identified only in some isolated areas at the edge of the large aggregates (Figure S4E and F), possessing average length and diameter equal to 2.3 ± 0.2 μm and 490 ± 50 nm, respectively (Figure S5A and B).

Conversely, the cured self-assembled SAMN-TiNT complexes resulted in a stable suspension, showing no sign of precipitation for at least six months. Furthermore, the synthetic product was observed using AFM (Figure 3A and B), showing a well-defined cylindrical shape. Indeed, the thickness of the anisotropic material is comparable to its width, suggesting that the item possesses a circular cross section and therefore an overall tubular geometry. DLS analysis further substantiated the differences between the two materials. The mean hydrodynamic size of the product obtained from the nanohybrids was 823 ± 2 nm. This is the result of the median between the length and the diameter of the anisotropic shape of the material as analyzed by AFM, which is therefore compatible with the presence of individual rods in suspension. Most importantly, colloidal stability was corroborated by a large negative zeta potential (ζ) equal to −40 ± 2 mV, with its conductivity being 60 mS cm^–1^, representing an unprecedented feature for a hollandite material, likely a heritage of the parent TiNTs. TEM analysis further confirmed the formation of a sub-micrometric material with good monodispersity and a well-defined, partially hollow cylindrical shape (Figure 3C and D). Average length and diameter measurements were 3.3 ± 0.4 μm and 520 ± 50 nm, respectively (Figure S5C and D). Noteworthy, these sharp cylindrical cavities were clearly recognizable in many analyzed items, nicely recalling a round-bottom laboratory tube (Figure 3E). Thus, the initial self-assembly reaction is a mandatory prerequisite to obtain such a peculiar material, endowed with an unprecedented size, morphological feature, and exceptional colloidal stability. In particular, the presence of cavities is expected to hold value in the context of the ever-developing field of hollow and core–shell nanomaterials^42^ and nanoreactors,^43,44^ where nanoconfined spaces are used to obtain products with specific dimensions,^45^ discover new materials,^46^ build nanosensors,^47^ or obtain highly efficient or highly selective reactivities.^48–52^ Figure 3F shows a scheme of the hollandite cavity as a nanoreactor through the confinement of active nanomaterials.

**Figure 3:**
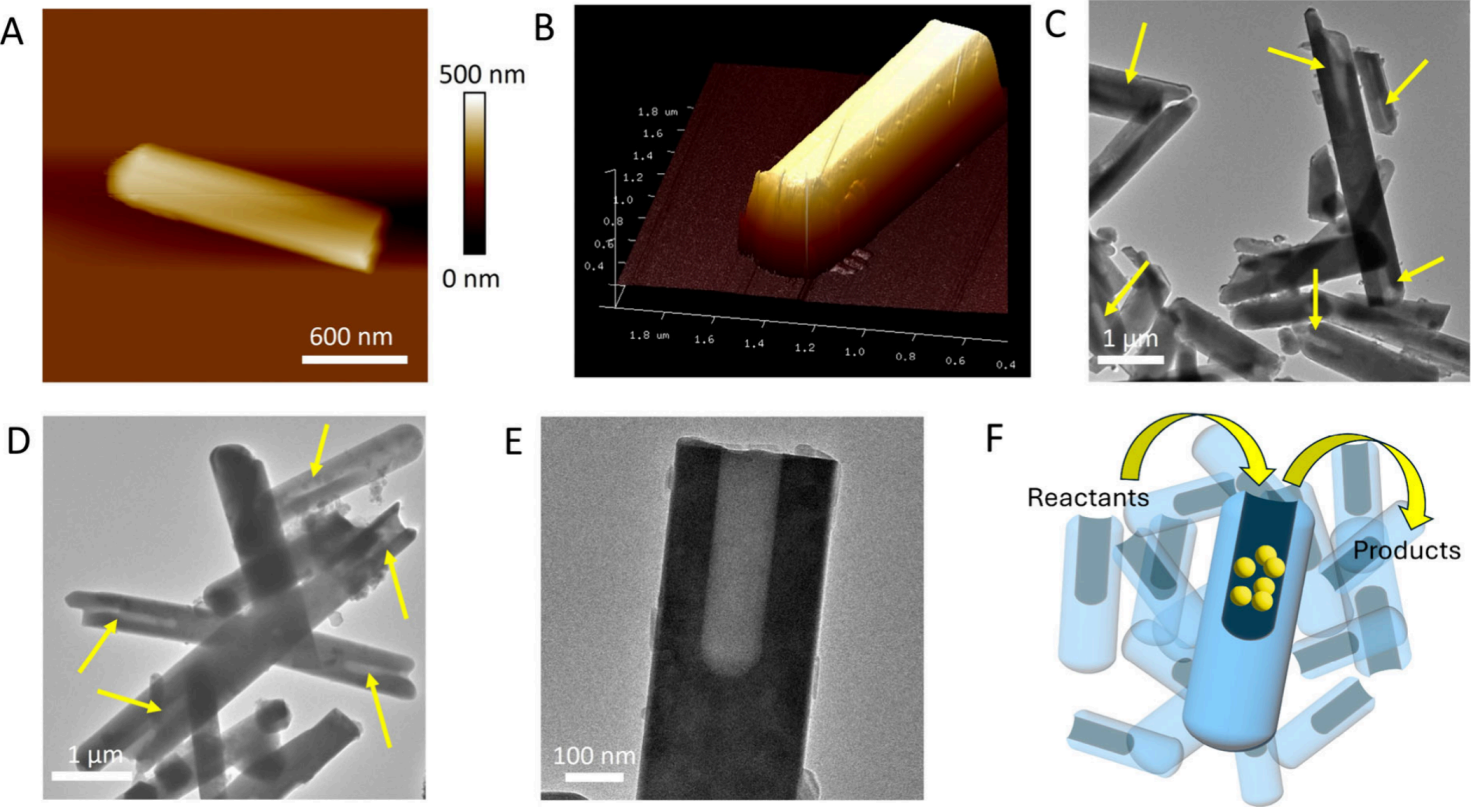
Morphological characterization of the synthetic products with the presynthetic hybridization step. (A and B) Atomic force micrographs showing a representative 2D and 3D image of a single tubular item of KFTO hollandite. (C and D) TEM-BF images of KFTO hollandite tubes, where the presence of cylindrical cavities in the nanosized range can be appreciated (indicated with yellow arrows). (E) TEM-BF image showing a single well-defined rod-like KFTO hollandite crystal with a sharp cylindrical cavity. (F) Schematic representation of a nanoreactor development using the preformed KFTO hollandite cavity.

An EDS-equipped STEM was used to analyze qualitatively the KFTO hollandite composition, and a representative object is reported in Figure 4A, while its corresponding EDS spectrum is shown in Figure 4B. Color-coded element representations of the same object shown in Figure 4A are reported in Figure 4C, demonstrating a homogeneous distribution of K, Fe, and Ti. In particular, the following KFTO average atomic composition fractions were calculated from the areas in Figure 4D, E, and F: K 6.6 ± 0.7%, Fe 5.5 ± 0.9%, Ti 26 ± 2%, and O 62 ± 2% (Table S4). Additional signals were collected from different sites of the sample, showing good agreement with expected hollandite supergroup chemistry.^28^ This further confirms the stoichiometry assessed by the XRPD analysis and, therefore, the proposed tetragonal symmetry, as previously described.^23^ In Figure 4D, E, and F, it is also possible to appreciate the rod cavities in different analysed items, as indicated by the yellow arrows.

**Figure 4:**
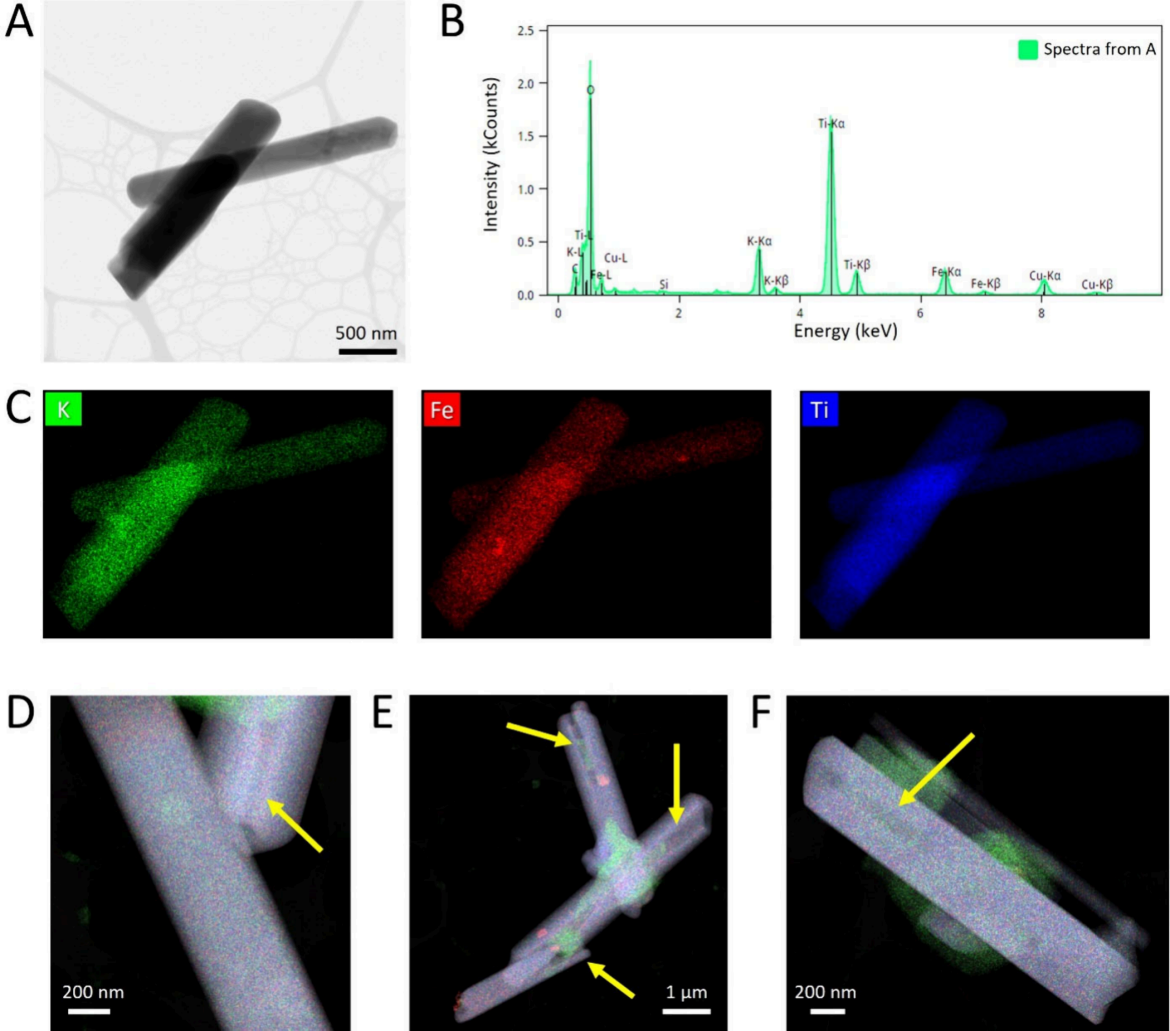
STEM-BF and EDS maps of KFTO hollandite crystals obtained by curing at 800 °C; hybrids self-assembled using 50 mM KCl, 100 mg L^–1^ of TiNTs, and 25 mg L^–1^ of SAMNs. (A) STEM image of KFTO hollandite tubes with the EDS-related spectrum shown in (B). (C) Elemental distributions (K+Ti+Fe+O) as individual maps (D) to (F) show composite EDS maps of different areas; the presence of the aforementioned cavities in the nanosized range can be appreciated as well (indicated with yellow arrows). K, Fe, and Ti are reported in green, red, and blue, respectively.

Figure 5 shows representative TEM bright images and diffractograms of the KFTO hollandite synthesized with an excess of maghemite, where it is possible to discriminate between KFTO hollandite and hematite crystals. Indeed, the sample is characterized by two morphologically distinct components, the aforementioned tubular KFTO hollandite structures and globular hematite nanoparticles (Figure 5A and B). The bottom section of the rod and its lattice fringes are visible in higher magnification HR-TEM images (Figure 5C and D, respectively). Figure 5E and F show the resulting diffractograms from the HR-TEM image, very close to a zone axis (for the zone axis determination method, see Supporting Information, Figures S2 and S6 and accompanying description). The *d*-spacing values reported in Figure 5F of 5.03 Å, 2.84 Å, 2.53 Å, 2.22 Å, 1.68 Å, and 1.44 Å are consistent with the presence of a hollandite structure *sensu.* ^28^ Hematite globular nanoparticles appear to be attached to the rod surface in a fashion similar to the native SAMNs-TiNTs complex, to some extent (Figure 5G, H, and I). This secondary nanosized material, displaying a diameter of approximately 50 nm, is attributed to the hematite as a contribution recorded by XRPD analysis (*vide supra*).

**Figure 5:**
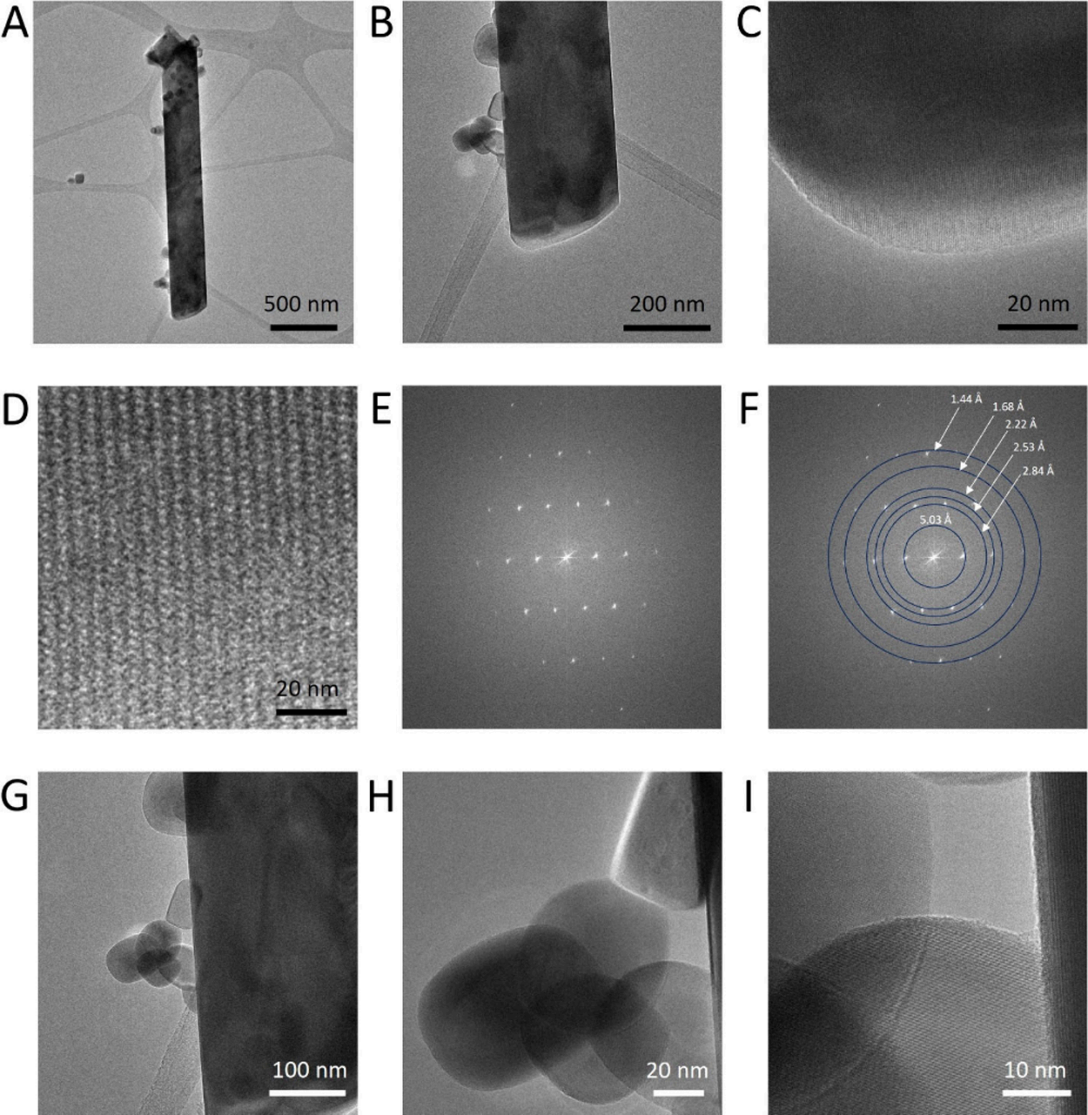
Maghemite nanoparticles excess influence on hollandite synthesis. In (A), the TEM-BF image of a KFTO hollandite tube surrounded by nanoparticles of hematite is shown; in (B, C), the same rod is shown at higher magnification. (D) shows a HR-TEM image of KFTO hollandite lattice fringes. In (E, F), diffractograms extracted from the HR-TEM image (C), with (F) including the *d*-spacing attributed to KFTO hollandite. In (G), (H), and (I), hematite nanoparticles in TEM-BF images at different magnifications.

In Figure S7, EDS maps of potassium, titanium, and iron are consistent with hematite as the byproduct. In particular, hematite is distinct from the titanium-based oxide in terms of its higher Fe content.

Alternatively, hematite was present in the form of larger nanoparticle aggregates adhering to KFTO hollandite rods, with the latter acting as nucleation sites for globular nanoparticles (Figure S7). EDS results showed the presence of K, Ti, Fe, and O as elemental components of the tubes, clearly ascribable to KFTO hollandite, whereas the chemical composition of the nanoparticle aggregates essentially consists of Fe and O, in a ca. 2:3 elemental ratio, attributed to the Fe_2_O_3_ counterpart (Table S5). Cu and C signals in the EDS spectrum come from the grid used to support the sample and the TEM holder. The tendency of hematite to nucleate on the surface of the KFTO hollandite rods is unavoidable. In any case, hematite only mildly affected the purity of the optimized synthetic product.

Concluding, a synthetic strategy was developed for the production of a hollandite material. The protocol consisted of a simple hydrothermal treatment of electrostatically stabilized binary hybrids comprising TiNTs and SAMNs. Optimized conditions involved a preliminary 2 h incubation step with 100 mg L^–1^ of TiNTs, 25 mg L^–1^ of SAMNs, and 50 mM KCl, followed by curing at 800 °C. A new single-crystalline KFTO hollandite was obtained, characterized by a sub-micrometer tube-like shape and the presence of nanosized cavities in both ends of their anisotropic structure. Such objects showed a large negative zeta potential (i.e., −40 mV) leading to stable colloidal suspensions. When compared to similar hydrothermally generated materials, the evolution into hollandite took place at a substantially lower temperature. The present study paves the way to a new generation of synthetic materials, exploiting nanomaterial hybrids as reagents.

## Supplementary Material



## Notes

All materials and instruments used in this study, synthesis details, additional characterizations, and supplementary figures (PDF)

## ABBREVIATIONS

TiNTs: titanate nanotubes
SAMNs: surface active maghemite nanoparticles
FTIR: Fourier-transform infrared spectroscopy
MRS: μ-Raman spectroscopy
AFM: atomic force microscopy
TEM: transmission electron microscopy
STEM: scanning electron transmissiom, microscopy
DLS: dynamic light scattering
XRPD: X-ray powder diffraction
KFTO: potassium, iron, titanium, oxygen
HR-TEM: high-resolution TEM
TEM-BF: TEM bright field
EDS: energy dispersive X-ray spectroscopy
JCPDS: Joint Committee on Powder Diffraction Standards

## References

[1] Taylor-Pashow K. M., Della Rocca J., Huxford R. C., Lin W. (2010). Hybrid nanomaterials
for biomedical applications. ChemComm..

[2] Li J., Lu W., Yang Y., Xiang R., Ling Y., Yu C., Zhou Y. (2023). Hybrid nanomaterials
for cancer immunotherapy. Adv. Sci..

[3] He C., Liu D., Lin W. (2015). Nanomedicine
applications of hybrid nanomaterials built
from metal–ligand coordination bonds: nanoscale metal–organic
frameworks and nanoscale coordination polymers. Chem. Rev..

[4] Yang X., Fan Y., Liang J., Cao R., Zhang B., Li J., Li Z., He S., Liu N., Du J., Hu Y. (2024). Polyaptamer-Driven
Crystallization of Alendronate for Synergistic Osteoporosis Treatment
through Osteoclastic Inhibition and Osteogenic Promotion. ACS Nano.

[5] Kaur S., Singh M., Brkljaca R., Anderson S. R., Korte J., Svoboda P., Mašková-Černá S., Urban S., Shukla R., Ramanathan R., Bansal V. (2024). Artificial Magnetosomes: Molecularly Restructured SPIONs
with Enhanced Potential for Magnetic Imaging. Mater. Today Chem..

[6] Gwyther R. E. A., Côté S., Lee C.-S., Miao H., Ramakrishnan K., Palma M., Dafydd Jones D. (2024). Optimising
CNT-FET Biosensor Design through Modelling of Biomolecular Electrostatic
Gating and Its Application to β-Lactamase Detection. Nat. Commun..

[7] Nalepa M.-A., Panáček D., Dědek I., Jakubec P., Kupka V., Hrubý V., Petr M., Otyepka M. (2024). Graphene Derivative-Based Ink Advances
Inkjet Printing Technology for Fabrication of Electrochemical Sensors
and Biosensors. Biosens Bioelectron.

[8] Hu S., Huang J., Arul R., Sánchez-Iglesias A., Xiong Y., Liz-Marzán L.
M., Baumberg J. J. (2024). Robust
Consistent Single Quantum Dot Strong Coupling in Plasmonic Nanocavities. Nat. Commun..

[9] Anand A., Unnikrishnan B., Wang C.-Y., Lai J.-Y., Lin H.-J., Huang C.-C. (2024). Phosphate Ester-Linked Carbonized
Polymer Nanosheets
to Limit Microbiological Contamination in Aquaculture Water. NPJ. Clean Water.

[10] Zhang S., Pelligra C. I., Feng X., Osuji C. O. (2018). Direct
Assembly
of hybrid nanomaterials and nanocomposites. Adv. Mater..

[11] Cecconello A., Cencini A., Rilievo G., Tonolo F., Litti L., Vianello F., Willner I., Magro M. (2024). Chiroplasmonic DNA
Scaffolded “Fusilli” Structures. Nano Lett..

[12] Li Z., Fan Q., Yin Y. (2022). Colloidal self-assembly approaches
to smart nanostructured
materials. Chem. Rev..

[13] Arnon Z. A., Piperno S., Redeker D. C., Randall E., Tkachenko A. V., Shpaisman H., Gang O. (2024). Acoustically Shaped DNA-Programmable
Materials. Nat. Commun..

[14] Moetakef P., Larson A. M., Hodges B. C., Zavalij P., Gaskell K. J., Piccoli P. M., Rodriguez E. E. (2014). Synthesis
and crystal chemistry of
microporous titanates. J. Solid State Chem..

[15] Michiue Y., Watanabe M. (1999). Atomistic simulation
study of k-hollandite: Ionic correlation
and dynamics of the linearly disordered solid. PRB.

[16] Yadav M. K., Kothari A. V., Gupta V. K. (2011). Preparation
and characterization
of bi- and trimetallic titanium based oxides. Dyes Pigm..

[17] Aubin-Chevaldonnet V., Caurant D., Dannoux A., Gourier D., Charpentier T., Mazerolles L., Advocat T. (2007). Preparation and characterization
of (Ba, Cs)­(M,Ti)_8_O_16_ (M = Al^3+^,
Fe^3+^, Ga^3+^, Cr^3+^, Sc^3+^, Mg^2+^) hollandite ceramics developed for radioactive
cesium immobilization. J. Nucl. Mater..

[18] Xu Y., Wen Y., Grote R., Amoroso J., Shuller
Nickles L., Brinkmann K. S. (2016). A-site compositional effects in Ga-doped
hollandite
materials of the form Ba_x_Cs_y_Ga_2x+y_Ti_8–2x‑y_O_16_: implications for
Cs immobilization in crystalline ceramic waste forms. Sci. Rep..

[19] Hassan Q. U. I., Yang D., Zhou J. P., Lei Y.-X., Wang J.-Z., Awan S. U. (2018). Novel single-crystal
hollandite K 1.46 Fe 0.8 Ti 7.2
O 16 microrods: synthesis, double absorption, and magnetism. Inorg. Chem..

[20] Cao C., Singh K., Kan W. H., Avdeev M., Thangadurai V. (2019). Electrical
properties of hollandite-type Ba_1.33_Ga_2.67_Ti_5.33_ O_16_, K_1.33_Ga_1.33_Ti_6.67_ O_16_, and K_1.54_Mg_0.77_Ti_7.23_ O_16_. Inorg. Chem..

[21] Hayashi F., Furui K., Shiiba H., Yubuta K., Sudare T., Terashima C., Teshima K. (2020). Flux Growth of Single-Crystalline
Hollandite-Type Potassium Ferrotitanate Microrods from KCl Flux. Front. Chem..

[22] Gorshkov N. V., Mikhailova D. A., Vikulova M. A., Gorbunov M. V., Gorokhovskii A. V. (2021). Electrochemical
properties of hollandite K_1.5_Fe_1.5_Ti_6.5_O_16_ with carbon coating. Russ. J.
Inorg. Chem..

[23] Tumurugoti P., Betal S., Sundaram S. K. (2021). Hollandites’
crystal chemistry,
properties, and processing: a review. Int. Mater.
Rev..

[24] Gorokhovsky A. V., Tretyachenko E. V., Goffman V. G., Gorshkov N. V., Fedorov F. S., Sevryugin A. V. (2016). Preparation
and dielectric properties of ceramics based
on mixed potassium titanates with the hollandite structure. Inorg. Mater..

[25] Gorshkov N., Vikulova M., Mikhail G., Mikhailova D., Burmistrov I., Kiselev N., Artyukhov D., Gorokhvsky A. (2021). Synthesis of the hollandite-like copper doped potassium
titanate high-k ceramics. Ceram. Int..

[26] Ebinumoliseh I., Grosvenor A. P. (2018). Effect of synthetic method and annealing temperature
on the structure of hollandite-type oxides. Inorg. Chem..

[27] Granados E., Martinez-Calderon M., Groussin B., Colombier J. P., Santiago I. (2024). Highly Uniform Silicon Nanopatterning with Deep-Ultraviolet
Femtosecond Pulses. Nanophotonics.

[28] Biagioni C., Capalbo C., Pasero M. (2013). Nomenclature tunings in the hollandite
supergroup. Eur. J. Mineral..

[29] Ramakrishna S., Mahender N., Reddy J. R., Kurra S., Nagabhushan E., Vithal M. (2015). Preparation and characterization
of nitrogen doped
K_2_M_2_Ti_6_O_16_ (M = Cr and
Fe) with enhanced photocatalytic activity. Indian
J. Chem..

[30] Liu W., Yi H., Zheng Q., Li X., Zhang H. (2017). Y-doped Na_3_V_2_(PO_4_)_2_F_3_ compounds
for sodium ion battery cathodes: electrochemical performance and analysis
of kinetic properties. J. Mater. Chem..

[31] Kong X., Wang X., Ma D., Huang J., Li J., Qin Y., Yin L., Feng Q. (2019). Hydrothermal synthesis and electrochemical
performance of K_0.8_Fe_0.8_Ti_1.2_O_4_ lithium ion battery anode. Mater. Lett..

[32] Lee J. L., Miller R. C., Moloney L. J., Prieto A. L. (2019). The development
of strategies foe nanoparticle synthesis: Consideration for deepening
understanding of inherently complex systems. J. Solid State Chem..

[33] Naciri Y., Ghazzal M. N., Paineau E. (2024). Nanosized tubular clay
minerals as
inorganic nanreactors for energy and environmental applications: A
review to fill current knowledge gaps. Adv.
Colloid Interface Sci..

[34] Lee J., Kim S. N., Lee I. S. (2014). Functionalization of hollow nanoparticles
for nanoreactor applications. Nano today..

[35] Zaera F. (2022). Designing
sires in heterogeneous catalysis:are we reaching selectives competitive
with thos of homogeneous catalyst?. Chem. Rev..

[36] Magro M., Molinari S., Venerando A., Baratella D., Zoppellaro G., Salviulo G., Zboril R., Vianello F. (2020). Colloidal
maghemite nanoparticles with oxyhydroxide-like interface and chiroptical
properties. Appl. Surf. Sci..

[37] Doebelin N., Kleeberg R. (2015). Profex: a graphical
user interface for the Rietveld
refinement program *BGMN*. J.
Appl. Crystallogr..

[38] Krishna S. R., Shrujana P., Palla S., Sreenu K., Velchuri R., Vithal M. (2015). Preraration, characterization
and photocatalytic studies
of K_2_Al_2_Ti_6_O_16_, K_2‑x_Ag_x_Al_2_ Ti_6_O_16_ and K_2_Al_2_Ti_6_O_16‑x_N_y_. Mater. Res. Express..

[39] Zhao M., Russel P., Amoroso J., Misture S., Utlak S., Besmann T., Shuller-Nickles L., Brinkman K. S. (2020). Exploring the links
between crystal chemistry, cesium retention, thermochemistry and chemical
durability in single-phase (Ba,Cs)_1.33_(Fe,Ti)_8_O_16_ hollandite. Mater. Sci..

[40] Zhao M., O’Quinn E., Birkner N., Xu Y., Lang M., Brinkman K. (2021). radiation
damage and thermal annealing in tunnel structured
hollandite materials. Acta Mater..

[41] Patel V. R., Agrawal Y. K. (2011). Nanosuspension:
an approach to enhance solubility of
drugs. J. Adv. Pharm. Technol. Res..

[42] Fan, Q. ; Lu, Y. ; Xu, S. ; Xu, G. ; Cai, Z. ; Feng, J. ; Wu, C. ; Brinzari, T. V. ; Pan, L. ; Yin, Y. Core-Shell Nanospheres with Controllable Zinc Ion Release for Time-Sensitive Steganography. Adv. Mater. Technol. 2023, 8 (18). 10.1002/admt.202300469.

[43] Swisher J. H., Jibril L., Petrosko S. H., Mirkin C. A. (2022). Nanoreactors for
Particle Synthesis. Nature Reviews Materials.

[44] Wang, Y. ; Xie, F. ; Zhao, L. Spatially Confined Nanoreactors Designed for Biological Applications. Small 2024, 20, 10.1002/smll.202310331. 38183369

[45] Jibril, L. ; Cheng, M. ; Wahl, C. B. ; Dravid, V. P. ; Mirkin, C. A. Polymer-Mediated Particle Coarsening within Hollow Silica Shell Nanoreactors. Chem. Mater. 2022, 34 (11), 5094 10.1021/acs.chemmater.2c00510.

[46] Liang, J. ; Chen, S. ; Ni, E. ; Tang, J. ; Cao, G. ; Wang, H. ; Li, Z. ; Zeng, M. ; Fu, L. High-Entropy Alloy Array via Liquid Metal Nanoreactor. Adv. Mater. 2024, 36 (31), 10.1002/adma.202403865. 38857624

[47] Chen, C. ; Fan, Q. ; Li, Z. ; Cai, Z. ; Ye, Z. ; Yin, Y. Colorimetric Pressure Sensing by Plasmonic Decoupling of Silver Nanoparticles Confined within Polymeric Nanoshells. Nano Lett. 2024, 24 (12), 3737 10.1021/acs.nanolett.4c00268. 38498412

[48] Tian, Q. ; Jing, L. ; Du, H. ; Yin, Y. ; Cheng, X. ; Xu, J. ; Chen, J. ; Liu, Z. ; Wan, J. ; Liu, J. ; Yang, J. Mesoporous Carbon Spheres with Programmable Interiors as Efficient Nanoreactors for H2O2 Electrosynthesis. Nat. Commun. 2024, 15 (1), 10.1038/s41467-024-45243-w. PMC10834542 38302469

[49] Zhao, S. ; Chen, Z. ; Zheng, Z. ; Luan, X. ; Gao, Y. ; Qi, L. ; Xue, Y. ; Li, Y. A Graphdiyne Nanoreactor for Conversion of NO3– to NH3 from Wastewater. Adv. Funct Mater. 2023, 33 (51), 10.1002/adfm.202308507.

[50] Wu, Y. ; Song, M. ; Zhao, Z. ; Wang, G. H. ; Wang, C. ; Astruc, D. Integrating Theory with the Nanoreactor Concept to Synthesize Hollow Carbon Sphere-Encapsulated PtNi Alloys for Enhanced H2 Generation. Carbon Energy 2024, 6 (7), 10.1002/cey2.455.

[51] Sinai N. G., Dones Lassalle C. Y., Kelm J. E., Patel S. K., Park S.-M., Tan M. J. H., Odom T. W., Dempsey J. L. (2024). Electrochemical
Control of Strong Coupling of CdSe Exciton-Polaritons in Plasmonic
Cavities. Nano Lett..

[52] Nankya, R. ; Xu, Y. ; Elgazzar, A. ; Zhu, P. ; Wi, T. ; Qiu, C. ; Feng, Y. ; Che, F. ; Wang, H. Cobalt-Doped Bismuth Nanosheet Catalyst for Enhanced Electrochemical CO _2_ Reduction to Electrolyte-Free Formic Acid. Angewandte Chemie International Edition 2024, 63 (36), 10.1002/anie.202403671. 38887161

