# Supplementary material for: Colloidal Hollandite Holey Rods Produced by Presynthetic Nanohybridization
Source: Nano Lett. 2025 Jun 26;25(32):12142–50. doi: 10.1021/acs.nanolett.5c01451 (PMC12356057; doi:10.1021/acs.nanolett.5c01451)
Supplement: Supplementary file 1 [file nl5c01451_si_001.pdf]

Supporting Information for

Colloidal Hollandite Holey Rods Produced by Pre-synthetic Nano-hybridization

Authors

Ilenia Maria D'Angeli[a1], Graziano Rilievo[b1], Simone Molinari[c], Anna Barbaro[a,d], Alessandro Cecconello[b,e], Aura Cencini[b], Federica Tonolo[b], Mary Bortoluzzi[b], Marco Favero[a], Andrea Basagni[f], Sheryl A. Singerling[d], Frank E. Brenker[d], Fabio Vianello[b], Massimiliano Magro[b*], Gabriella Salviulo[a]

[a]*Department of Geosciences, University of Padova, Via* Gradenigo 6, 35131 Padova*, Italy*

[b]*Department of Comparative Biomedicine and Food Science, University of Padova, Viale dell'Università 16, 35020, Legnaro (PD), Italy*

[c]*Museum of Nature and Humankind, Mineralogy Section Alessandro Guastoni, University of Padua, Via Giotto 1, 35121 Padua, Italy*

[d]Schwiete Cosmochemistry Laboratory, Department of Geosciences, Goethe University Frankfurt, Altenhöferallee 1, 60438 Frankfurt, Germany

[e] Department of Molecular and Translational Medicine, University of Brescia, Viale Europa, 11, 25123, Brescia, Italy

[f]Department of Chemical Sciences, University of Padova, Via Marzolo 1, 35131 Padova, Italy

[1]*These authors contributed equally.*

***corresponding author**

Materials and Methods

Optimization of the synthetic procedure

Titanate nanotubes (TiNTs) and surface active maghemite nanoparticles (SAMNs) were synthesized and re-suspended according to the respective protocols reported elsewhere[1-2-3]. The here proposed protocol consisted of the formation and curing of a TiNTs-SAMNs composite. In order to optimize the KFTO hollandite synthetic pathway, the products of the self-assembly wet reaction and the following heat treatment were investigated using constant 100 mg L^{-1} TiNTs and varying KCl (50, 20, 10, 5, 1 mM) as well as SAMNs (0, 25, 50, 100, 250, 500 mg L^{-1}) concentrations.

The two nanomaterials were hybridized exploiting their opposite zeta potentials through a simple self-assembly approach. Thus, after the preparation of TiNTs and SAMNs colloidal suspensions, the samples underwent an incubation phase for 2 hours under agitation at room temperature. After the incubation, in order to evaluate the stability of the binding and eliminate loosely bound nanotubes, the as-obtained electrostatically stabilized hybrids were isolated by means of an external magnet and subjected to several washing steps. The washing steps included the incubation of magnetically responsive material for 15 minutes under agitation at room temperature with a KCl solution at a concentration equal to the one used in the incubation phase (*vide supra*), followed by the asportation of the supernatant with the aid of a magnet (Figure S1).

The concentration of bound TiNTs was estimated as the difference between the incubation concentration and the concentration of nanotubes lasting in the aqueous milieu after incubation and released in the washing solutions. For quantification purposes, the concentration of the free diamagnetic nanomaterial was monitored through UV-Vis spectrophotometry. In particular, a calibration curve was built according to the Lambert-Beer law, by plotting TiNT maximum of absorbance at 245 nm against increasing concentrations of the tubular nanomaterial in the range comprised between 0.75 and 6 mg L^{-1}. The extrapolated extinction coefficient of 0.19 L mg^{-1}, was

used to estimate unbound TiO_2 nanotubes. For this purpose, all supernatants, after the self-assembly reaction as well as the washing solutions, devoid of magnetic materials, were collected and analyzed via UV-Vis spectrophotometry. After the wet reaction the magnetically isolated hybrids were characterized and underwent a stage of curing. Two temperatures of 600°C and 800°C were compared.

The resulting powders were analyzed with Fourier Transform Infrared Spectroscopy (FTIR), micro-Raman Spectroscopy (MRS), Dynamic Light Scattering (DLS), X-ray powder diffraction (XRPD), and Transmission Electron Microscopy (TEM) and Scanning Transmission Electron Microscopy (STEM).

Instrumentation

Magnetic separations were carried out using Nd-Fe-B magnets (N35, 263–287 kJ/m3 BH, 1170–1210 mT flux density by Power magnet—Germany). Optical absorption spectra were acquired using a Cary 60 spectrophotometer (Agilent Technologies, CA, USA) and quartz cuvettes (1 cm light path). FTIR absorption spectra were recorded in 400–4000 cm^{-1} wavelength range using an IRAffinity-1S spectrometer (Shimadzu Corp., Japan). Results were analyzed by LabSolutions IR software (Shimadzu Corp., Japan). Raman spectra were acquired using a Witec alpha 300 R (Oxford Instruments, UK) equipped with Zeiss microscopy (10x, 20x, 50x LD, 50x, 100x). The instrument is equipped with a 532 nm excitation laser source, two spectrometers (UHTS 600 (VIS) and UHTS 400 (NIR)) and a motorized x-y-sample scanning stage for confocal Raman imaging. Results were processed and analyzed by RamanCrystalHunter software and database[4].

Zeta-potential and size-distribution were measured in water by dynamic light scattering (DLS) using a Zetasizer Nanoparticle analyzer ZEN3600 (Malvern Instrument, Malvern, UK). LogNormal-function was used to obtain statistical analysis on the size-distribution. XRPD was performed in reflective mode using a Philips X'Pert PRO diffractometer instrument equipped with an X'Celerator detector.

Analyses were carried out in Bragg Brentano geometry using a Cobalt anode. The standard reflection measurements included the following conditions from 3° to 88.5° of 2 theta with a step of 0.017° and an acquisition time of 100s per step. The Rietveld method for structure refinement, as implemented in Profex-BGMN v.5.2.5, was run on scans taken from 10° to 140° of 2 theta, a step of 0.008° and an acquisition time of 150s per step, for a total of 5 h and 11 minutes of measurement. The incident beam optics included a divergence fixed slit of 1/4°, an anti-scatter slit of 1°, and a soller slit of 0.04 rad. The software used was Profex-BGMN v.5.2.5[5].

The morphology and microstructure of the nanohybrids were characterized by TEM and high-angle annular dark-field (HAADF) scanning transmission electron microscopy (HAADF-STEM) using a STEM JEOL F200 operated at 200 kV. Elemental analysis and mapping were performed using a JEOL 100 mm^2 silicon drift energy dispersive X-ray spectrometer (EDX). A carbon supported copper grids, 400 mesh size, were used for preparation of the sample. A Thermo Scientific (TS) Talos F200-X G2 STEM was used at the Schwiete Cosmochemistry Laboratory at Goethe University, Frankfurt. The STEM images were acquired with a TS Ceta-S 4k x 4k 16M camera. Scanning Transmission Electron Microscope (STEM) high-angle annular dark field (HAADF) and bright field (BF) images were collected with a collection angle range of 58-200 mrad. Energy dispersive X-ray spectroscopy (EDS) data were collected using four windowless TS EDS silicon drift detectors. All Talos STEM data was processed using the TS Velox software.

For phase identification, we used a combination of compositional information from EDS analyses as well as fast-Fourier-transforms (FFTs) data. In particular, we ran FFT on high-resolution TEM bright field images, a technique that generates diffractograms. We compared the measured *d*-spacings of the FFTs to theoretical values available in the literature using simulated patterns in the SingleCrystal software.

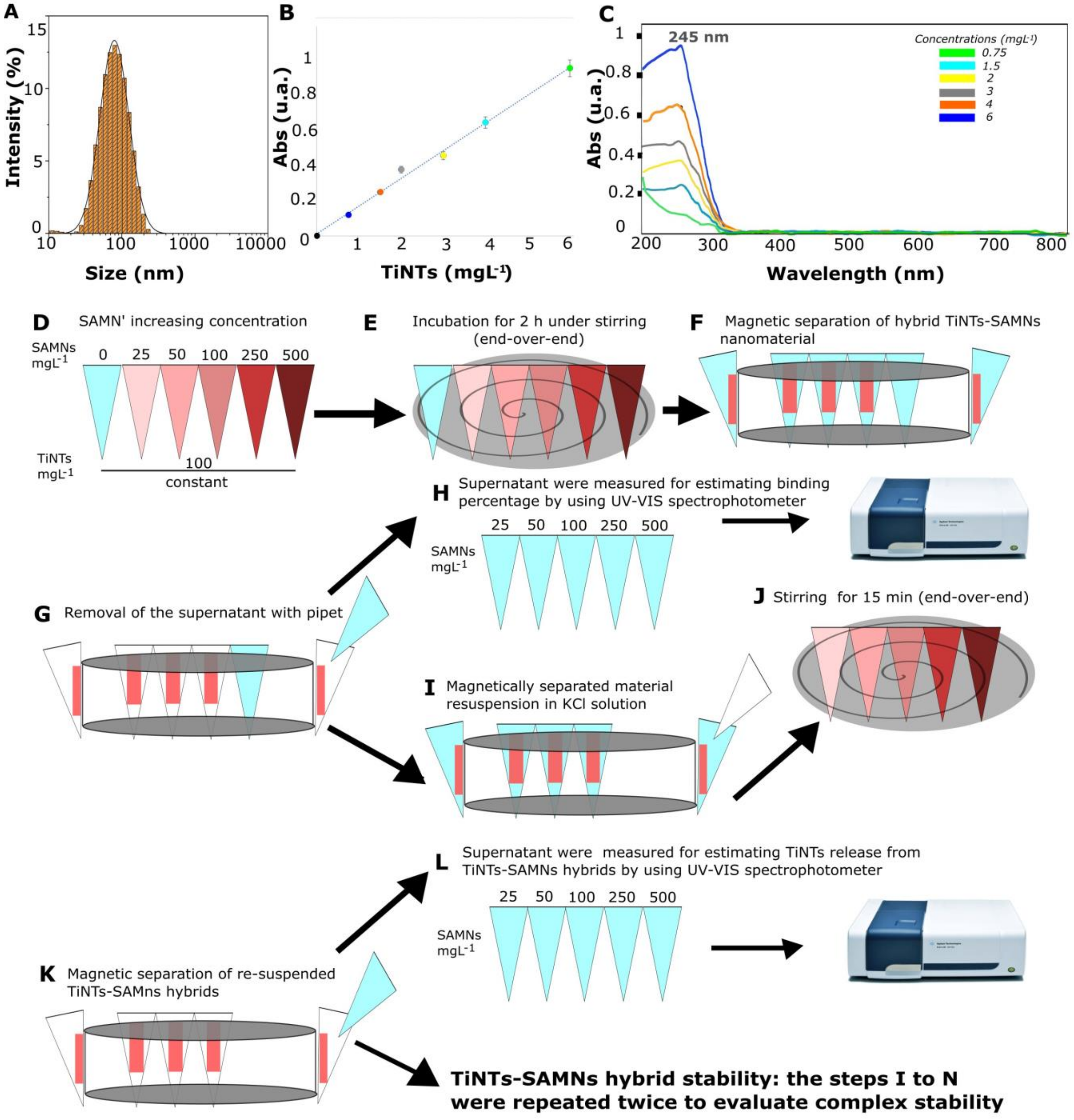

Figure S1. Scheme of KFTO synthesis optimization. (A) TiNT size measured with DLS. (B). Calibration curve according to Lambert-Beer law e concentration of TiNTs. (C) The peak at 245 nm, typical of TiNTs, decreases with the lowering of the concentration. (D) to (L) Steps of the procedure to functionalize TiNTs with SAMNs in a salty solution with fixed KCl.

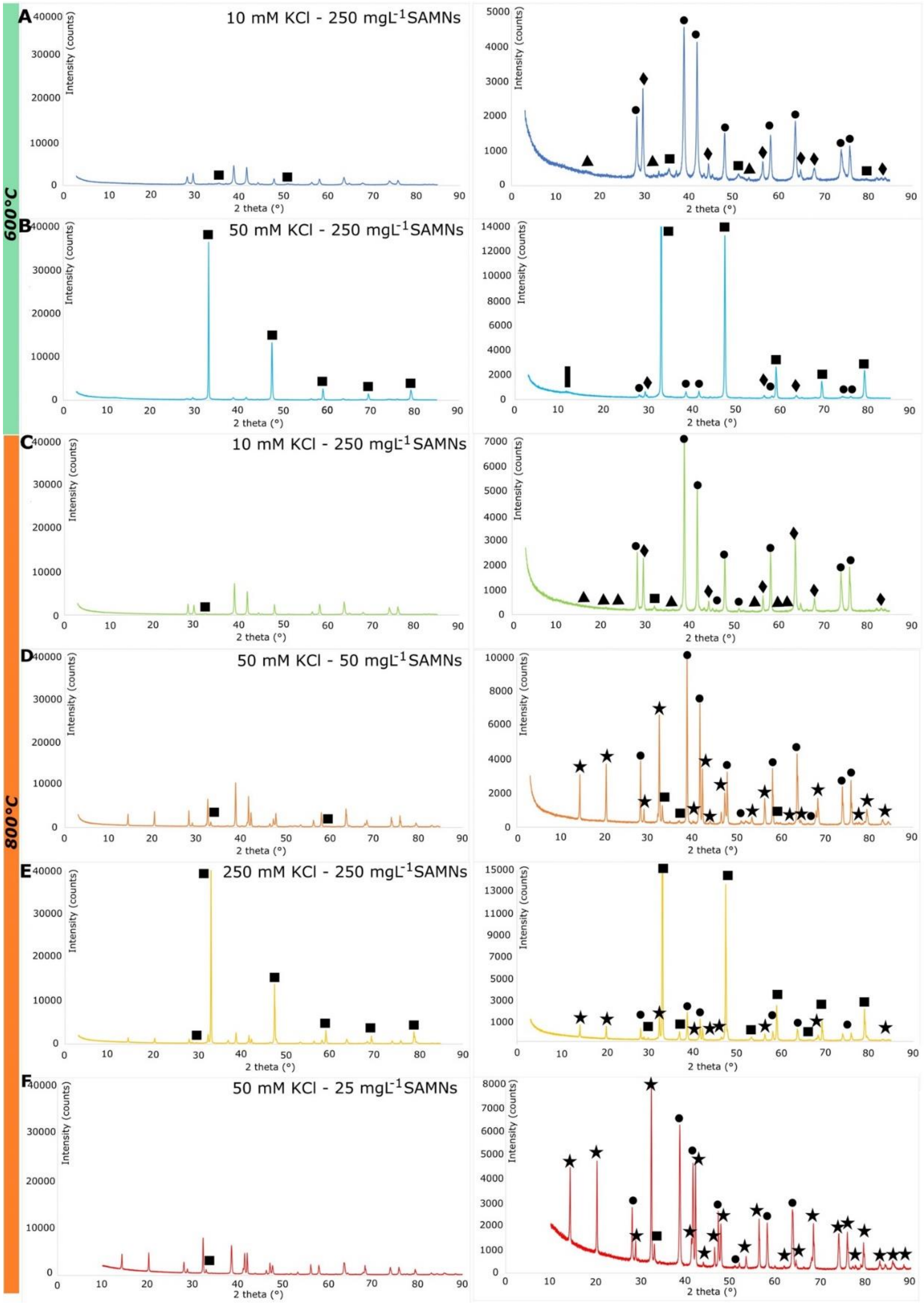

Figure S2. XRPD patterns of synthetic products obtained at different KCl (10, 50 and 250 mM), SAMNs concentrations (25, 50, 250 mg L^{-1}) and at two different at reaction temperatures (600 and 800°C). In the left column the diffractograms are shown at the same intensity scale (y-axis) displaying with a square the peak of sylvite, whilst on the right, their magnifications are reported. The diamond shape represents anatase, the square sylvite and halite, the triangle freudenbergite and pseudobrookite, the rectangle is for TiO_2 nanotubes, the circle is hematite, and the star is KFTO hollandite.

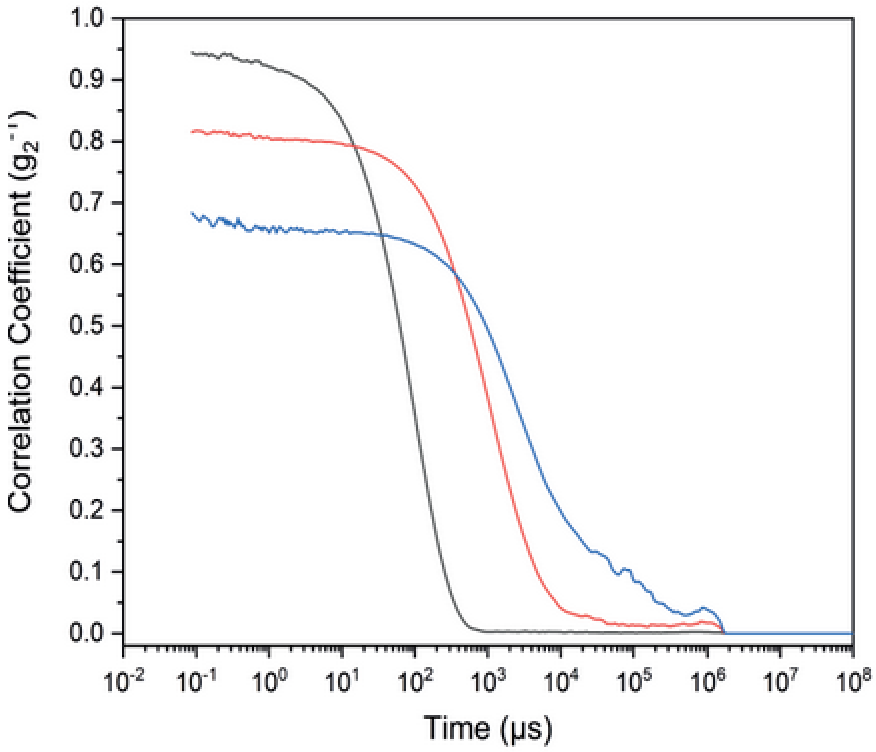

Figure S3. Correlation curve obtained by DLS analysis of TiNTs (black), KFTO (red) and cured SAMNs-TiNTs without functionalization (blue).

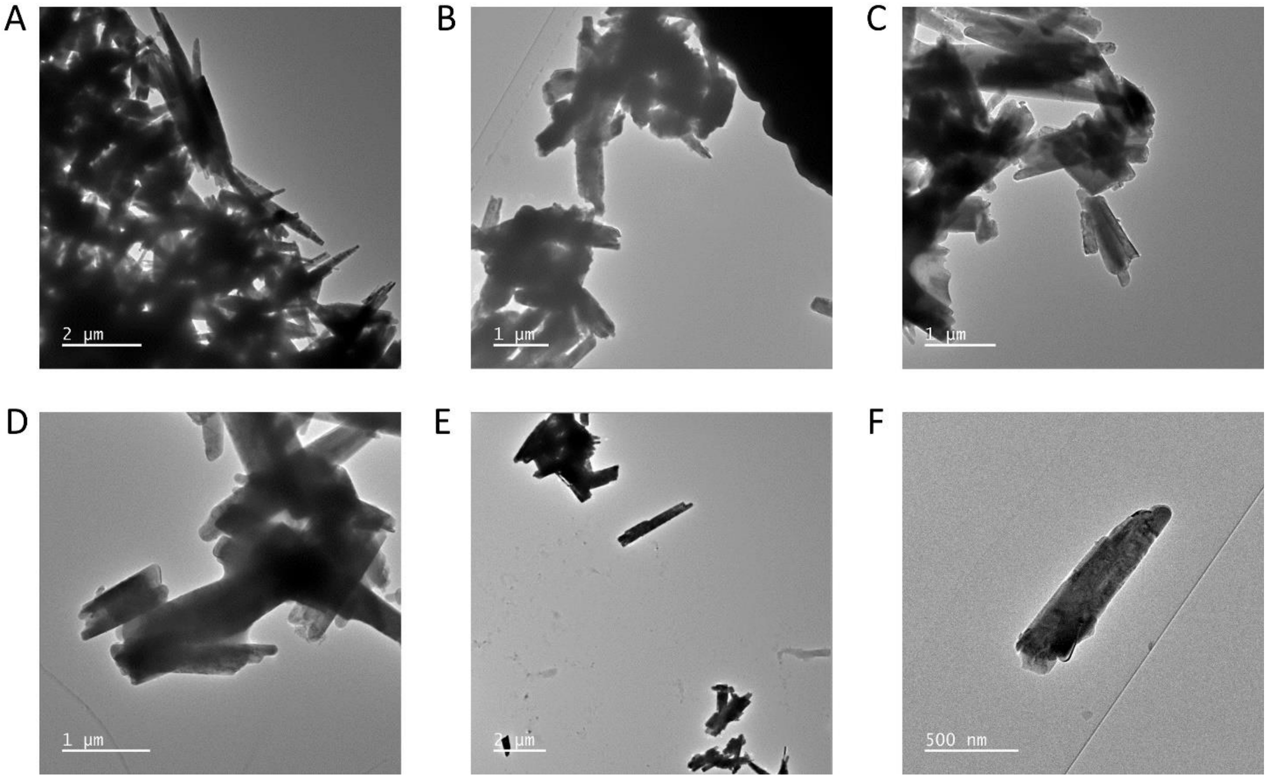

Figure S4. TEM morphological characterization of the reaction products in the absence of the pre-synthetic hybridization. Extended bundled aggregates (A); morphological disordered and dramatically entangled anisotropic items (B and C). Undefined matrix merging the objects together (D); irregular columnar shapes (E and F).

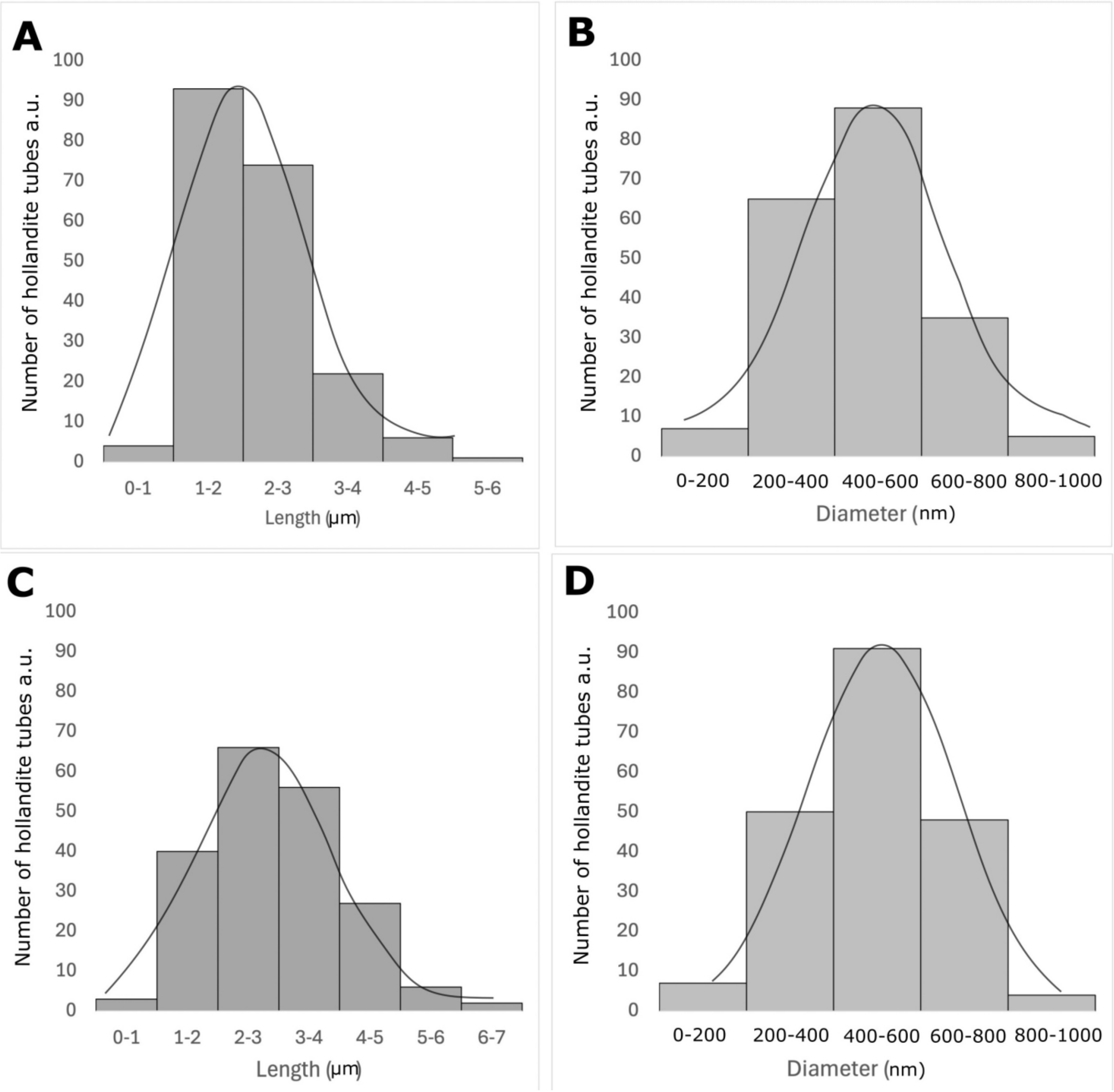

Figure S5. TEM size comparison of the synthetic products in the absence (A, B) and in the presence (C, D) of the preliminary TiO_2- Fe_2O_3 nano- hybridization after curing at 800°C. In the left column are reported the item lengths while in the right column their diameters.

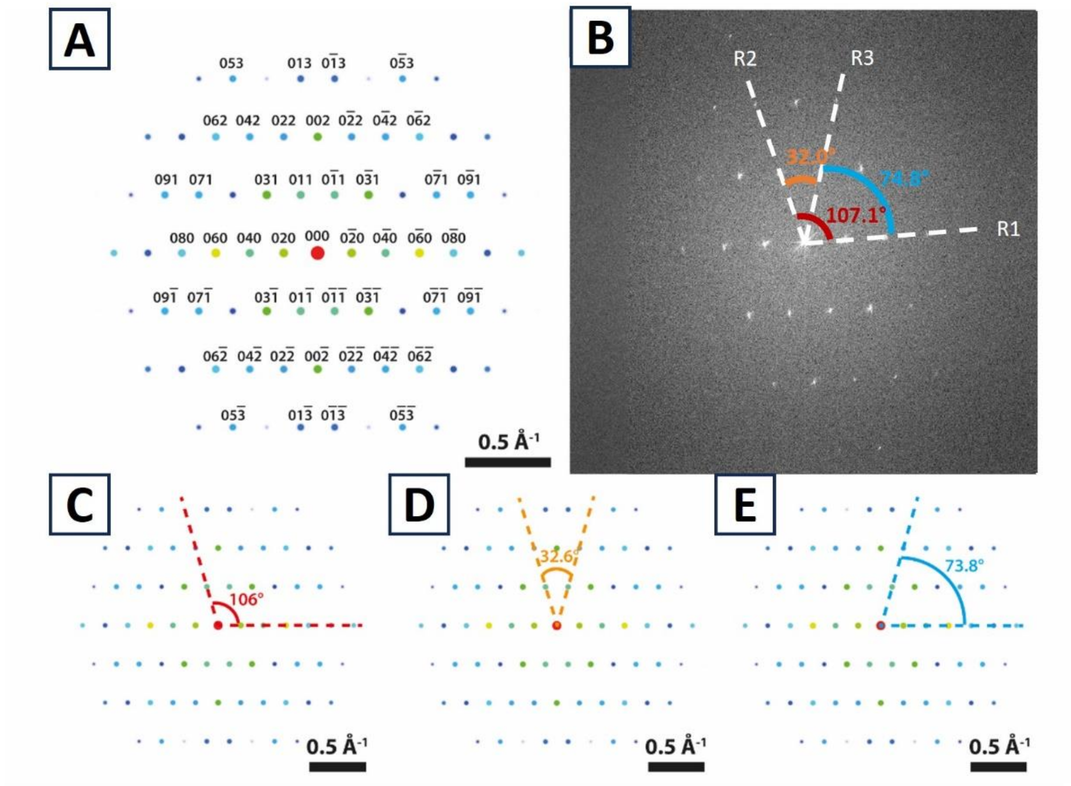

F

	Measured		Theoretical	[1 0 0]
	Å	hkl	d spacing	%diff
R1	5.000	0 -2 0	5.070	1.4%
R2	2.755	0 1 1	2.847	3.3%
R3	2.755	0 -1 1	2.847	3.3%
	°		Angle	%diff
R1^R2	107.1		106.0	1.03%
R1^R3	74.8		73.8	1.35%
R2^R3	32.0		32.6	1.85%

Figure S6. KFTO hollandite crystal lattice characterization by TEM: (A) Hollandite SingleCrystal software simulated pattern down [1 0 0]; (B) shows the measured d spacings and angles from the diffractogram, whereas (C), (D), and (E) show the simulated pattern angle measurements. The measured data best match Hollandite as viewed down [1 0 0]. (F) measured and theoretical values used for the zone axis determination.

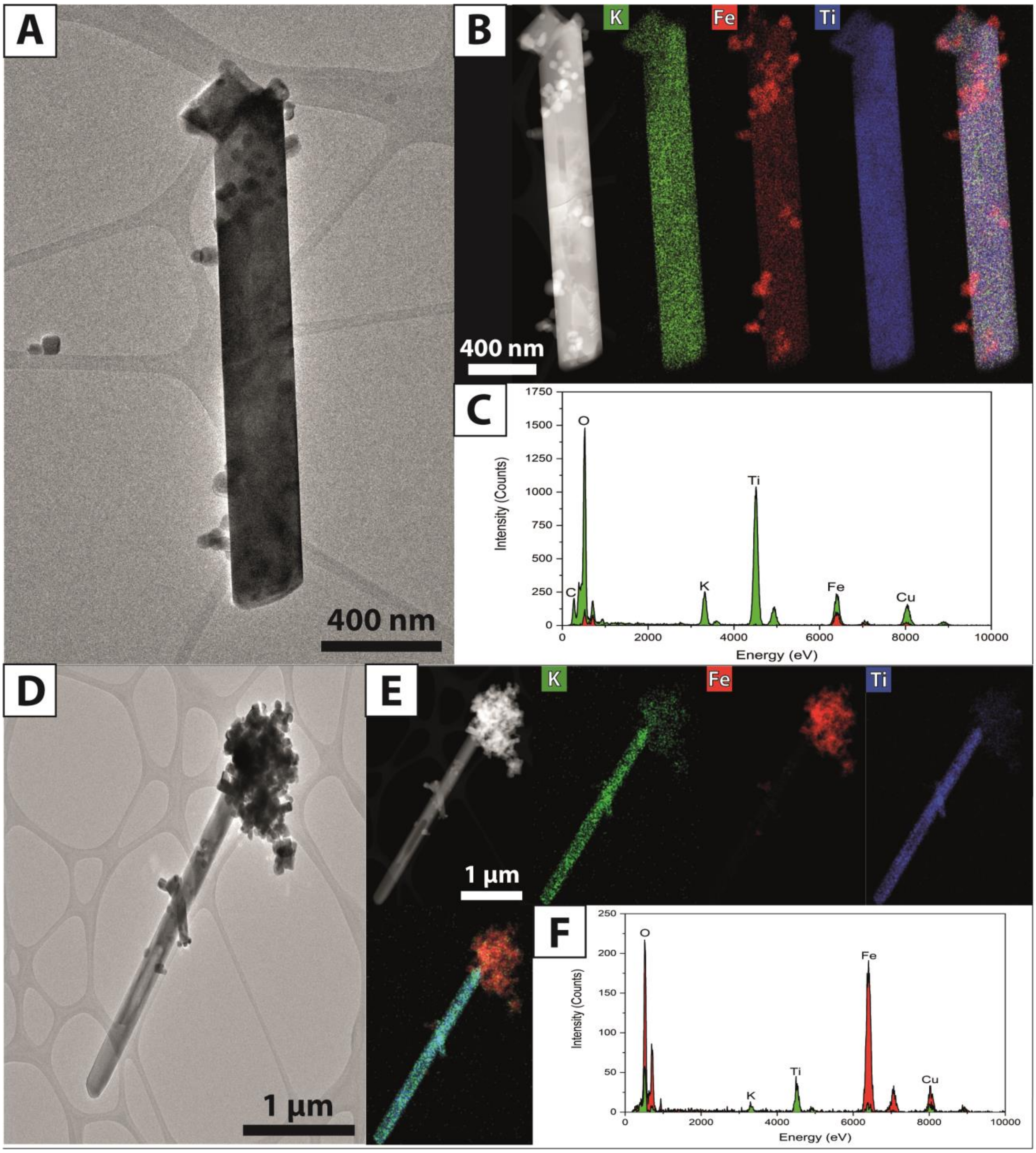

Figure S7. Two hollandite-hematite particles with EDS maps and their spectra. (A) the TEM bright field (BF) image of a single KFTO hollandite tube with hematite particles. (B) HAADF-STEM image together with the EDS elemental maps and composite (RGB = Fe-K-Ti) of hollandite and hematite. (C) Representative EDS spectra of hollandite (in green) and hematite (in red) from the particle shown in (B). (D) TEM BF image of two hollandite tubes with a globular hematite aggregate. (E) HAADF-STEM image together with the EDS elemental and composite (RGB = Fe-K-Ti) maps of hollandite and hematite. (F) Representative EDS spectra of hollandite (in green) and hematite (in red) for the particle shown in (E).

Table S1. Percentage of binding of TiNTs through magnetic separation after incubation with increasing concentrations of KCl and SAMNs.

	SAMN (mg L^{-1})				
**KCl (mM)**	**5**	**50**	**100**	**250**	**500**
1	0	0	0	0	0
5	0	0	0	14	0
10	80.7	91.4	84.2	100	100
20	80.6	90.7	90.7	99.3	98.1
50	90.4	100	99.8	100	100

Table S2. List of the samples treated at 600°C and 800°C. For each temperature the concentration of KCl and SAMNs are reported, and their crystalline-phases percentage obtained after calcination.

T (°C)	KCl (mM)	SAMNs (mgL^{-1})	Hollandite %	Hematite %	Sylvite %	Halite %	Anatase %	Rutile %	Freudenbergite %	Pseudobrookite %	Jeppeite %	α-Fe %	TiO_2 nanotubes	Maghemite %
**600**	10	250	-	64	4	-	18	-	5	-	-	-	2	7
	50	250	-	8	80	-	3	-	-	-	-	-	4	5
**800**	10	250	-	83	-	-	11	2	2	2	-	-	-	-
	50	250	36	59	2	3	-	-	-	-	-	-	-	-
	50	100	56	33	-	-	-	-	-	-	-	11	-	-
	50	50	78	3	16	3	-	-	-	-	-	-	-	-
	50	25	75	4	16	3	2	-	-	-	-	-	-	-
	250	250	16	24	57	3	-	-	-	-	-	-	-	-
	250	100	4	2	92	2	-	-	-	-	-	-	-	-
	50	25*	75	4	20	1	-	-	-	-	-	-	-	-
	50	0	-	-	64	2	9	-	-	-	25	-	-	-

*synthesis without initial hybridization step.

Table S3. Rietveld refinement results for KFTO hollandite obtained after calcination at 800°C

lattice	a=b= 1.01503 (1) nm<br>c= 2.9717(1) nm;<br>α, β, γ=90°			
	Occupancy	x	y	z
Fe	0.24(3)	0.6489(1)	0.1683(1)	0
Ti(1-Fe)	0.76	0.6489(1)	0.1683(1)	0
O1	1	0.1667(3)	0.5415(3)	0
O2	1	0.1547(3)	0.7980(3)	0
K1	0.202(5)	0	0	0.7300(6)
K2	0.42(1)	0	0	0.5
R_{wp}	6.27	X^2	1.7	

Table S4. Elemental compositions obtained by EDS in the areas reported in Figure 4 D, E and F.

Panel D

Hollandite Element	Atomic fraction	Atomic error	Mass fraction	Mass error	Fit error
-	%	%	%	%	%
**O**	64.20	2.79	37.48	2.84	2.23
**K**	5.58	0.96	7.96	1.42	0.59
**Ti**	24.14	3.13	42.17	4.24	0.10
**Fe**	6.08	0.81	12.39	1.70	0.23

Panel E

Hollandite Element	Atomic fraction	Atomic error	Mass fraction	Mass error	Fit error
-	%	%	%	%	%
**O**	62.38	2.82	35.77	2.75	2.22
**K**	6.73	1.20	9.44	1.73	5.62
**Ti**	24.53	3.18	42.08	4.28	1.69
**Fe**	6.35	0.87	12.71	1.80	3.65

Panel F

Hollandite Element	Atomic fraction	Atomic error	Mass fraction	Mass error	Fit error
-	%	%	%	%	%
**O**	60.34	3.08	34.08	2.91	3.07
**K**	7.33	1.25	10.11	1.80	0.30
**Ti**	28.13	3.48	47.54	4.36	0.20
**Fe**	4.20	0.58	8.27	1.22	0.50

Table S5. Elemental compositions obtained by EDS in the two different areas of the aggregates shown in Figure S7 D and E.

Hematite Element	Atomic fraction	Atomic error	Mass fraction	Mass error	Fit error
-	%	%	%	%	%
**O**	63.58	3.02	36.33	3.14	2.61
**Fe**	36.42	3.02	63.67	3.14	0.68

Hematite Element	Atomic fraction	Atomic error	Mass fraction	Mass error	Fit error
-	%	%	%	%	%
**O**	60.32	3.25	30.34	2.87	2.77
**Fe**	39.68	3.25	69.66	2.87	0.40

References

(1) Zennaro, L.; Magro, M.; Vianello, F.; Rigo, A.; Mariotto, G.; Giarola, M.; Froner, E.; Scarpa, M. Stable acqueous solution of naked titanate nanotubes. *ChemPhysChem*, **2013**, 14, 2786-2792.

(2) Magro, M.; Molinari, S.; Venerando, A.; Baratella, D,; Zoppellaro, G.; Salviulo, G.; Zboril, R.; Vianello, F. Colloidal maghemite nanoparticles with oxyhydroxide-like interface and chiroptical properties. *Appl. Surf. Sci*. **2020**, 534, 147567.

(3) Molinari, S.; Magro, M.; Baratella, D.; Salviulo, G.; Ugolotti, J.; Filip, J.; Tucek, J.; Zoppellaro, G.; Zboril, R.; Vianello, F. Smart synthetic maghemite nanoparticles with unique surface properties encode binding specificity toward As^{III}. *Sci. Total Environ.* **2020**, 1(741), 140175.

(4) Nestola, F.; Zhang, Q.; Day, M.C.; Lorenzon, S.; Pamato, M.G.; Rocchetti, I.; Bendazzoli, C.; Novella, D.; Mazzoli, C.; Sassi, R.; Pearson, D.G.; Smith, E.M.; Scott, M.; Barbaro, A.; Brenker, F.E.; Santello, L.; Molinari, S.; Qu, K.; Wang, Y.; Škoda, R.; Alvaro, M.; Gilio, M.; Murri, M.; Kasatkin, A.V. *RamanCrystalHunter*: a new program and database for processing, analysis, and identification of Raman spectra. *Am. Min.* **2024**

(5) Doebelin, N.; Kleeberg, R. Profex: a graphical user interface for the Rietveld refinement program *BGMN. J. Appl. Crystallogr.* **2015***,* 29(48), 1573-1580.
